# Associations between the size of individual plantar intrinsic and extrinsic foot muscles and toe flexor strength

**DOI:** 10.1186/s13047-022-00532-9

**Published:** 2022-03-21

**Authors:** Yuki Kusagawa, Toshiyuki Kurihara, Sumiaki Maeo, Takashi Sugiyama, Hiroaki Kanehisa, Tadao Isaka

**Affiliations:** 1 Graduate School of Sport and Health Science, Ritsumeikan University, 1-1-1 Noji Higashi, Kusatsu, Shiga 525-8577 Japan; 2 Research Organization of Science and Technology, Ritsumeikan University, Kusatsu, Shiga Japan; 3 Faculty of Sport and Health Science, Ritsumeikan University, Kusatsu, Shiga Japan

**Keywords:** Maximal anatomical-cross sectional area, Muscle volume, Stepwise multiple linear regression analysis, Adductor hallucis oblique head, Toe grip strength

## Abstract

**Background:**

The size of the plantar intrinsic and extrinsic foot muscles has been shown to be associated with toe flexor strength (TFS). Previous studies adopted the size of limited plantar intrinsic foot muscles or a compartment containing several muscles as an independent variable for TFS. Among the plantar intrinsic and extrinsic foot muscles, therefore, it is unclear which muscle(s) primarily contributes to TFS production. The present study aimed to clarify this subject.

**Methods:**

In 17 young adult men, a series of anatomical cross-sectional area of individual plantar intrinsic and extrinsic foot muscles was obtained along the foot length and the lower leg length, respectively, using magnetic resonance imaging. Maximal anatomical cross-sectional area (ACSA_max_) and muscle volume (MV) for each constituent muscle of the plantar intrinsic foot muscles (flexor hallucis brevis; flexor digitorum brevis, FDB; abductor hallucis; adductor hallucis oblique head, ADDH-OH; adductor hallucis transverse head, ADDH-TH; abductor digiti minimi; quadratus plantae) and extrinsic foot muscles (flexor hallucis longus; flexor digitorum longus) were measured. TFS was measured with a toe grip dynamometry.

**Results:**

TFS was significantly associated with the ACSA_max_ for each of the ADDH-OH (r = 0.674, *p* = 0.003), ADDH-TH (r = 0.523, *p* = 0.031), and FDB (r = 0.492, *p* = 0.045), and the MV of the ADDH-OH (r = 0.582, *p* = 0.014). As for the ADDH-OH, the correlation coefficient with TFS was not statistically different between ACSA_max_ and MV (*p* = 0.189). Stepwise multiple linear regression analysis indicated that ACSA_max_ and MV of the ADDH-OH alone explained 42 and 29%, respectively, of the variance in TFS.

**Conclusion:**

The ADDH-OH is the primary contributor to TFS production among the plantar intrinsic and extrinsic foot muscles as the result of the stepwise multiple linear regression analysis.

## Introduction

Toe flexor strength (TFS) plays an important role in postural control during standing [1] and walking [2]. TFS is positively associated with the performance of sports-related activities in adolescents and young adults [3–5], whereas older adults with a low level of TFS have impairments of activities of daily living tasks [6–9]. Moreover, an intervention of a training program aiming to enhance toe muscular strength improves jumping height in young adults [10] and mobility in older adults [11]. Thus, identifying influential factors for TFS will provide useful information for designing a training program aiming to enhance physical performance in various populations.

TFS is generated by the combined activities of plantar intrinsic and extrinsic foot muscles [12]. Some of the plantar intrinsic foot muscles (i.e., flexor hallucis brevis, FHB and flexor digitorum brevis, FDB) are specialized in toe flexion, while others (e.g., abductor hallucis, ABH and adductor hallucis) mainly act on toe adduction/abduction [13]. Among the extrinsic foot muscles, only the flexor hallucis longus (FHL) and flexor digitorum longus (FDL) are specialized in toe flexion [13]. On the other hand, the great toe alone can produce TFS twice as great as the lesser toes can [14], the latter of which is about one-third of the TFS produced by all toes [15]. Considering these, the magnitude of TFS may primarily depend on the force generation capacity of the muscles that mainly act on great toe flexion (i.e., FHB and FHL).

It is known that the anatomical cross-sectional area (ACSA) [14–17] and muscle thickness [14] of plantar intrinsic or extrinstic foot muscles, determined by using ultrasonography or magnetic resonance imaging (MRI), significantly associates with TFS. Moreover, Kurihara et al. [17] revealed through stepwise multiple linear regression analysis that the ACSA of medial parts of plantar intrinsic foot muscles was a major determinant for TFS production. However, these studies have quantified the size of limited plantar intrinsic foot muscles [14–16] or a compartment containing several plantar intrinsic foot muscles [17] as an independent variable for examining the association with TFS. Thus, the anatomical function of individual plantar intrinsic foot muscles has not been considered for examining the relationship between TFS and muscle size, and therefore little information is available from previous findings as to which muscle(s) primarily contributes to the magnitude of TFS.

Furthermore, the ultrasonographic studies cited above [14–16] have determined ACSA or muscle thickness from a single image in which the thickest region of muscle belly was visually identified. In addition, Kurihara et al. [17] measured the ACSA of a compartment containing several plantar intrinsic foot muscles from a single MR image at 80% of the foot length (FL), because the ACSA of the entire plantar intrinsic foot muscle was largest at the corresponding position [18]. However, muscle volume (MV) and maximal anatomical cross-sectional area (ACSA_max_) have been shown to be more strongly associated with joint torque [19, 20] or strength [20] compared to ACSA and muscle thickness. Nevertheless, no studies have determined MV or ACSA_max_ of individual plantar intrinsic muscles and examined its association with TFS.

The purpose of the present study was to elucidate the muscle(s) that primarily contributes to TFS production. To this end, this study examined the association of ACSA_max_ and MV of each constituent muscle of the plantar intrinsic and extrinsic foot muscles with TFS, and then conducted stepwise multiple liner regression analysis by using TFS as a dependent variable with the ACSA_max_ and MV values of the muscles as independent variables. We hypothesized that the muscles that mainly act on great toe flexion, i.e., the FHB and FHL, would be selected as the primary contributors to TFS among the individual plantar intrinsic and extrinsic foot muscle.

## Methods

### Participants

Seventeen healthy young men (age, 21.4 ± 1.9 yrs.; height, 171.0 ± 5.9 cm; body mass, 62.3 ± 5.8 kg; mean ± standard deviation), with no history of a diagnosed neuromuscular disorder or lower limb injury, voluntarily participated in this study. This study was approved by the Ethics Committee of Ritsumeikan University. All participants provided prior written informed consent based on the guidelines of the Declaration of Helsinki.

### Experimental procedure

In this study, all participants firstly attended morphological measurements (body height and body mass). Then, MRI measurements were conducted to obtain the foot and lower leg images. After the completion of morphological and MRI measurements, TFS was determined by using a toe grip dynamometer.

### Measurements

#### Muscle size variables (ACSA_max_ and MV)

T1-weighted MR images of foot and lower leg were acquired using a 1.5 T (Signa HDxt, GE Healthcare UK Ltd., Buckinghamshire) and 3.0 T (Magnetom Skyra, Siemens Healthcare, Erlangen) MR system. Of all participants in this study, eleven participants were scanned by 1.5 T MRI, and the others (six participants) were scanned by 3.0 T MRI, due to the update of the MR system in Ritsumeikan university. To acquire the whole foot image, the participants lay in a supine position on the examination table of the MR systems with their dominant foot and ankle encased in the ankle coils (1.5 T MR system: HD Knee/Foot coil, GE Healthcare UK Ltd., Buckinghamshire; 3.0 T MR system: Foot/Ankle coil, Siemens Healthcare, Erlangen). To reduce motion artifacts during image acquisition, the foot and ankle were positioned in the coils and stabilized with Velcro straps so that the ankle was kept at an angle of 90 degrees of ankle plantarflexion (neutral position). Serial whole foot images were acquired from the sesamoids and calcaneal tuberosity of foot perpendicular to the plantar aspect of the foot, using a fast spin-echo sequence in 1.5 T MR system according to Chang et al. [18] (repetition time = 500 ms, echo time = 13 ms, slice thickness = 4 mm, gap between slices = 0 mm, field of view = 120 × 120 mm, flip angle = 90 degrees, matrix = 512 × 512) and 3.0 T MR system (repetition time = 700 ms, echo time = 12 ms, slice thickness = 3.5 mm, gap between slices = 0 mm, field of view =125 × 125 mm, flip angle =120 degrees, matrix = 1024 × 1024). The data acquisition time for foot was approximately 9 min. For the lower leg image, participants lay on the examination table and lower legs were placed parallel to the main magnetic field. Serial cross-sectional lower leg images were acquired from the knee cleft to just proximal to the malleoli (1.5 T MR system: repetition time = 600 ms, echo time = 7.7 ms, slice thickness = 10 mm, gap between slices = 0 mm, field of view = 360 × 360 mm, flip angle = 90 deg, matrix = 256 × 256; 3.0 T MR system: repetition time = 700 ms, echo time = 9.4 ms, slice thickness = 5 mm, gap between slices = 0 mm, field of view = 360 × 360 mm, flip angle = 120 deg, matrix = 1024 × 1024). The data acquisition time for each scan was approximately 3 min.

**Fig. 1:**
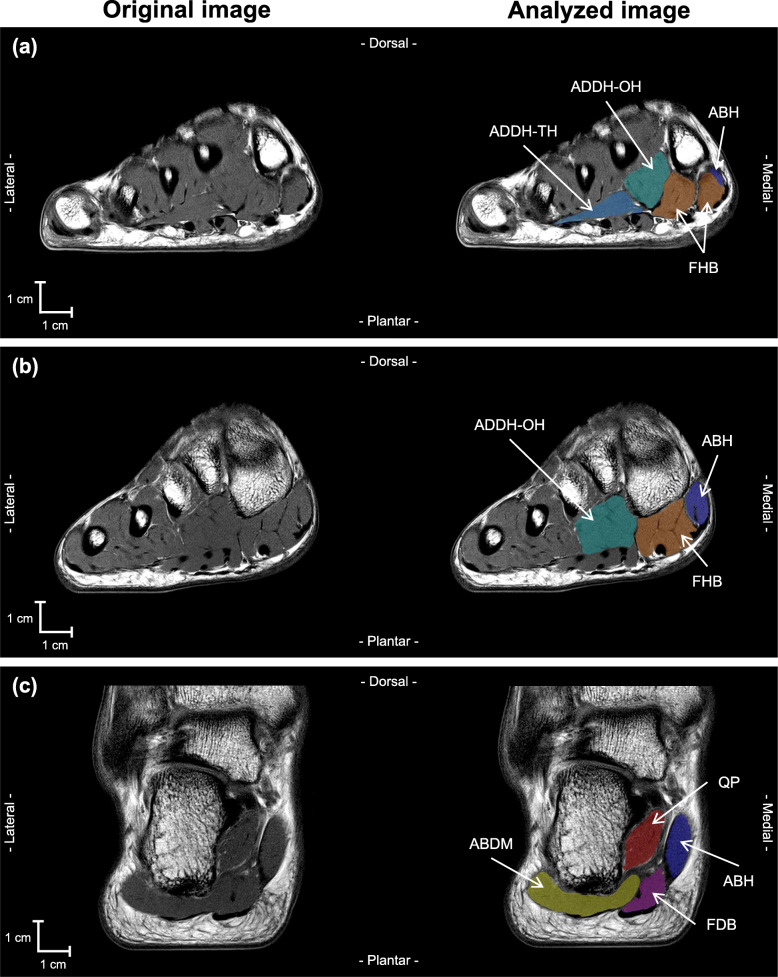
MRI images of right foot. Original and analyzed MR images are shown at the left and right side, respectively. Panel **a**, **b**, and **c** indicate MRI cross-sectional images obtained at the level of the mid-shaft of the first metatarsal of the foot, tarsometatarsal (Lisfranc) joint, sustentaculum tail, respectively. ABDM (abductor digiti minimi), ABH (abductor hallucis), ADDH-OH (adductor hallucis oblique head), ADDH-TH (adductor hallucis transverse head), FDB (flexor digitorum brevis), FHB (flexor hallucis brevis), QP (quadratus plantae) were manually segmented

Whole foot and lower leg images were analyzed by using specially designed image analysis software (SliceOmatic 5.0Rev-3b, Tomovision Inc., Montreal). Seven plantar intrinsic foot muscles and two extrinsic foot muscles were separately segmented by one examiner (YK): FHB, FDB, ABH, adductor hallucis oblique head (ADDH-OH), adductor hallucis transverse head (ADDH-TH), abductor digiti minimi (ABDM) and quadratus plantae (QP), as plantar intrinsic foot muscles, and FHL and FDL as extrinsic foot muscles. The segmentation was manually performed in every image from the most proximal to the most distal image in which the muscle was visible (Fig. 1). Non-contractile tissues such as bone, tendon, fat, connective tissue, nerve tissue, and blood vessels were carefully excluded. Other plantar intrinsic foot muscles (e.g., lumbrical and flexor digiti minimi) were excluded from the analysis because these muscles could not be visually separated from each other.

Foot length (FL) was determined as the distance between the medial calcaneal tuberosity and sesamoids bone of the first metatarsal in MR images [18]. The positions of all ACSAs of each intrinsic foot muscle were expressed relative to FL (0% FL: medial calcaneal tuberosity, 100% FL: sesamoids bone of the first metatarsal). Furthermore, lower leg length (LL) was determined as the distance between the most prominent point of the medial malleolus at the tibia and intercondylar eminence of tibia in MR images. The ACSAs of each extrinsic foot muscle were expressed relative to LL (0% LL: most prominent point of medial malleolus, 100% LL: intercondylar eminence of tibia).

Two variables, ACSA_max_ and MV, were adopted as representing the size of each muscle and muscle groups (explained below). ACSA_max_ was defined as the maximal ACSA along the FL (or LL), and muscle volume (MV) was calculated by summing all the ACSAs for each muscle multiplied by the slice thickness. Intra-rater repeatability for measuring ACSA_max_ and MV of four participants in this study was assessed by intra-class correlation coefficients (ICC). The ICC (1, 3) values of ACSA_max_ and MV of individual muscles were 0.869–0.999 and 0.842–0.996, respectively, and good to excellent repeatability was confirmed [21]. In addition, ACSA_max_ and MV of four muscle groups were analyzed: whole plantar intrinsic foot muscles (all analyzed plantar intrinsic foot muscles), extrinsic toe flexors (FHL and FDL), intrinsic great toe flexors (FHB, ABH, ADDH-OH, and ADDH-TH), and intrinsic lesser toes flexors (QP, FDB, and ABDM).

#### TFS

In accordance with the procedure adopted in a previous study [22], the maximum voluntary isometric TFS was measured using a commercially available isometric dynamometer (T.K.K. 3361, Takei Scientific Instrument Co., Niigata). The participants were seated in a chair and positioned the hip and knee joints at 90 degrees of flexion with the ankle joint at 90 degrees of dorsiflexion (neutral position) for generating TFS. The participant’s foot was placed on the dynamometer with the posterior heel adjusted at the heel stopper, and the first proximal phalangeal gripped the grip bar. During the measurement, the participants were instructed to cross their arms in front of their chest and gripped the grip bar using all toes as much as possible without any extraneous movements. Familiarization trials for 2–3 times with submaximal force outputs were conducted before the actual measurements. After participants completed the familiarization trials and a rest period of three minutes, the participants performed the task with maximal effort for at least 3 s. The maximal trial was repeated twice with at least one-minute rest, and the larger value of the two measurements was used for further analysis. The ICC for the two measurements was 0.869.

### Statistical analysis

Descriptive data are presented as means ± SDs. The ACSAs for each constituent muscle of plantar intrinsic and extrinsic foot muscles along the FL and LL, respectively, were calculated at 5% intervals by spread sheet software (Microsoft Excel, Microsoft Corp., Redmond, WA) and expressed relative to the FL and LL, respectively. Normality of measured variables was assessed by the Shapiro-Wilk test. MV of the ABDM was the only variable that was not normally distributed, and it was log-transformed for further analysis. All subsequent analysis was conducted by using parametric statistical tests. Pearson’s correlation coefficients were computed to examine the relationship between muscle size and TFS. When either or both of ACSA_max_ and MV had a significant correlation with TFS, the differences between these correlation coefficients for TFS in that muscle were statistically assessed by an online resource (http://comparingcorrelations.org [23] was used to implement Meng et al.’s [24] z test (two dependent groups, overlapping, and two-tailed test)). Stepwise multiple liner regression analysis was conducted by using TFS as a dependent variable, with ACSA_max_ or MV of the muscles that were significantly correlated with TFS as independent variables. The level of significance was set at *p* < 0.05. All data were analyzed using statistical software (SPSS 27.0, IBM Co., USA) unless otherwise stated.

## Results

### ACSA_max_ and MV of the individual plantar intrinsic and extrinsic foot muscles

FL, LL, and TFS were 15.4 ± 0.8 cm, 35.4 ± 1.8 cm, and 148.1 ± 35.0 N, respectively. Descriptive data on ACSA_max_ and MV for each of the plantar intrinsic foot muscles are summarized in Table 1. Among the analyzed plantar intrinsic foot muscles, the ACSA_max_ was the largest in the ADDH-OH, followed by the FHB, ABH, ABDM, FDB, QP, and ADDH-TH. The MV was the largest in the ABH followed by the FDB, ABDM, ADDH-OH, FHB, QP, and ADDH-TH. Descriptive data on ACSA_max_ and MV of the extrinsic foot muscles are summarized in Table 2. The ACSA_max_ and MV for the FHL were larger than those of FDL.

**Table 1:** ACSA_max_ and MV in each of the plantar intrinsic foot muscles

	ACSA_max_ (cm^2^)			MV (cm^3^)		
	Mean	±	SD	Mean	±	SD
	(Range)			(Range)		
Whole plantar intrinsic foot muscles	9.14	±	1.31	92.64	±	15.95
	(6.61–11.42)			(63.82–126.59)		
Intrinsic great toe flexors	7.04	±	1.27	48.46	±	8.69
	(4.43–9.01)			(33.36–68.58)		
FHB	3.05	±	0.65	12.44	±	2.55
	(1.94–4.65)			(7.60–17.12)		
ABH	3.01	±	0.55	21.76	±	4.58
	(2.26–4.10)			(15.35–32.41)		
ADDH-OH	3.42	±	0.59	12.76	±	2.27
	(2.19–4.40)			(8.08–18.06)		
ADDH-TH	1.01	±	0.29	1.51	±	0.54
	(0.50–1.64)			(0.79–2.90)		
Intrinsic lesser toes flexors	6.20	±	0.90	44.17	±	8.21
	(4.32–7.89)			(30.42–58.02)		
QP	1.97	±	0.44	11.49	±	2.54
	(1.35–2.88)			(7.76–16.51)		
FDB	2.48	±	0.41	16.62	±	3.58
	(1.76–3.19)			(10.07–23.16)		
ABDM	2.90	±	0.55	16.06	±	2.92
	(1.95–3.85)			(9.73–19.94)		

*ABDM* abductor digiti minimi, *ABH* abductor hallucis brevis, *ACSA*_*max*_ maximal anatomical cross-sectional area, *ADDH-OH* adductor hallucis oblique head, *ADDH-TH* adductor hallucis transverse head, *FDB* flexor digitorum brevis, *FHB* flexorhallucis brevis, *MV* muscle volume, *QP* quadratus plantae

**Table 2:** ACSA_max_ and MV in each of the extrinsic foot muscles

=		ACSA_max_ (cm^2^)			MV (cm^3^)		
		Mean	±	SD	Mean	±	SD
		(Range)			(Range)		
	Extrinsic toe flexors	5.33	±	0.89	88.60	±	13.74
		(4.10–7.02)			(72.02–114.57)		
	FHL	4.74	±	0.65	67.93	±	13.01
		(3.63–5.63)			(49.37–94.51)		
	FDL	1.55	±	0.22	20.66	±	3.22
		(1.20–2.00)			(15.76–27.06)		

*ACSA*_*max*_ maximal anatomical cross-sectional area, *FHL* flexor hallucis longus, *FDL* flexor digitorum longus, *MV* muscle volume

### Associations of ACSA_max_ or MV with TFS

Pearson’s correlation coefficients between TFS and ACSA_max_ or MV of individual muscles or muscle groups are summarized in Table 3. TFS was positively correlated with the ACSA_max_ for each of the ADDH-OH, ADDH-TH, and FDB, and also with the MV of the ADDH-OH. The magnitudes of the correlation coefficients with TFS were not statistically different between ACSA_max_ and MV (i.e., the r value of ACSA_max_ and TFS vs that of MV and TFS) in any muscles of the ADDH-OH, ADDH-TH, and FDB (z = − 1.443 – − 0.562, *p* = 0.149–0.599). The ACSA_max_ of the intrinsic great toe flexors was positively associated with TFS, but those of the whole plantar intrinsic foot muscle, intrinsic lesser toes flexors, and extrinsic toe flexors were not (Table 3). Moreover, MV of any other muscle groups was not significantly correlated with TFS.

**Table 3:** Correlations coefficients between TFS and ACSA_max_ or MV of the individual muscles or muscle groups

	ACSA_max_			MV		
	r		p	r		p
Plantar Intrinsic foot muscles	0.361		0.154	0.385		0.127
Intrinsic great toe flexors	0.604	*	0.010	0.454		0.067
FHB	0.442		0.076	0.430		0.085
ABH	0.364		0.151	0.277		0.282
ADDH-OH	0.674	**	0.003	0.582	*	0.014
ADDH-TH	0.523	*	0.031	0.474		0.054
Intrinsic lesser toes flexors	0.244		0.345	0.267		0.300
QP	0.115		0.660	0.126		0.631
FDB	0.492	*	0.045	0.271		0.293
ABDM	0.366		0.149	0.310		0.226
Extrinsic toe flexors	0.401		0.111	0.376		0.137
FHL	0.333		0.191	0.399		0.112
FDL	0.106		0.687	−0.009		0.974

Significance of Pearson’s correlations coefficients is indicated as follows: *p < 0.05, **p < 0.01. *ABDM* abductor digiti minimi, *ABH* abductor hallucis brevis, *ACSA*_*max*_ maximal anatomical cross-sectional area, *ADDH-OH* adductor hallucis oblique head, *ADDH-TH* adductor hallucis transverse head, *FDB* flexor digitorum brevis, *FDL* flexor digitorum longus, *FHB* flexor hallucis brevis, *FHL* flexor hallucis longus, *MV* muscle volume, *QP* quadratus plantae

Stepwise multiple liner regression analysis using ACSA_max_ as independent variable selected the ACSA_max_ of the ADDH-OH as an explainable factor for TFS: TFS(N) =10.61 × [ACSA_max_ of ADDH-OH (cm^2^)] + 40.26 (adjusted R^2^ = 0.418). Similarly, using MV as an independent variable selected the MV of the ADDH-OH as an explainable factor for TFS: TFS(N) =8.94 × [MV of ADDH-OH (cm^3^)] + 34.04 (adjusted R^2^ = 0.294).

## Discussion

The present study is the first case that determined the ACSA_max_ and MV for each constituent of the plantar intrinsic and extrinsic foot muscles and examined their associations with TFS. The major findings obtained here were that 1) TFS was significantly correlated with the ACSA_max_ of the ADDH-OH, ADDH-TH, and FDB, as well as with the MV of the ADDH-OH, and 2) the ACSA_max_ and MV of the ADDH-OH alone explained 42 and 29%, respectively, of the variance in TFS by stepwise multiple liner regression analysis. These results indicate that among the plantar intrinsic and extrinsic foot muscles, the ADDH-OH primarily contributes to TFS production.

At the start of the present study, we hypothesized that the muscle(s) mainly act on great toe flexion would be primarily contribute to the magnitude of TFS. However, the current results refuted this. The reason why the ADDH-OH was selected as the primary contributor to TFS may be attributable to a unique characteristic of this muscle having multiple functions of adduction and flexion of the great toe. The participants in this study were asked to grasp a solid straight bar for the TFS measurement. Toe grasping is often observed in the plantar grasp reflex, which is one of the primitive reflexes and consists of the combination of flexion and adduction of the toes [25]. Taken together, the toe grasping on the toe grip dynamometer for producing TFS can be considered as a complex multiple movement consisting of flexion and adduction rather than simple flexion of the toes. Therefore, the ADDH-OH, which acts on both adduction and flexion of the great toe, appears to contribute to TFS production more strongly than the muscles specialized in great toe flexion (i.e., FHB and FHL).

It is worth noting that among the plantar intrinsic and extrinsic foot muscles, not only the ADDH-OH but also ADDH-TH acts on both the flexion and adduction of the toes [13]. However, the ADDH-TH was not selected as the determinant for TFS. This may be due to the morphological differences between the two muscles. First, the ACSA_max_ and MV of the ADDH-OH were about 2.4 and 7.5 times, respectively, larger than those of the ADDH-TH (Table 1). Second, the ADDH-OH runs along the longitudinal direction of the foot and contracts closely parallel to the direction of the great toe movement during TFS production. On the other hand, the running direction of the ADDH-TH is perpendicular to the longitudinal axis of the foot, and this muscle contracts transversely and orthogonally to the direction of the great toe movement during TFS production. Thus, it is likely that the morphological features of the ADDH-OH would be suited for producing TFS more than that of the ADDH-TH, and consequently the ADDH-OH alone might have been selected as the contributor for TFS.

In general, force generation capacity of a muscle is theoretically best related to its physiological cross-sectional area (PCSA) [26]. Thus, adopting PCSA rather than ACSA_max_ and MV as independent variables would be desirable to examine the association of muscle size with TFS. However, the determination of PCSA in vivo needs data concerning pennation angle and fascicle length in addition to MV [19], and the procedure determining the PCSA of foot muscles has not been established likely due to their complex architecture. On the other hand, a previous study revealed that the correlation coefficients of ACSA_max_ and MV with muscle strength are comparable to that of PCSA [20, 27]. In the current results as well, both ACSA_max_ and MV of the ADDH-OH were selected as explainable factors for TFS, without a significant difference between ACSA_max_ and MV in their correlation coefficients with TFS. Thus, this study supports the previous studies [20, 27] and recommends future studies adopt either ACSA_max_ or MV as a representative muscle size index for examining the association between muscle size and TFS in vivo.

This study has some limitations relating to the procedure adopted for the TFS determination. We used a toe grip dynamometer to measure TFS, as this is one of the most commonly used devices [3–6, 8, 9, 15, 17] with its reliability confirmed [22]. Alternatively, other studies have measured TFS using a hand-held dynamometer [16] or plantar pressure platform [14]. Soysa et al. [28] reviewed the methods for measuring plantar intrinsic foot muscle strength and pointed out that the toe action during TFS production differs depending on devices and may change the activation of plantar intrinsic and extrinsic foot muscles. Thus, there is a possibility that the muscle(s) that primarily contributes to TFS production may differ from that identified here when another device is used to determine TFS. In addition, the participants examined here were asked to adjust their first proximal phalangeal at the solid straight grip bar and to exert maximal TFS using all toes, in accordance with the procedure of a previous study [22]. However, not all participants could grasp the grip bar with all toes because the length of each toe is usually different from each other and also different among participants. Thus, we cannot exclude the possible influence of the shape of individual participants’ toes on TFS measurements. Further study is needed to elucidate whether we could generalize the current results on the primary contributor to TFS production widely to various measurement devices and/or toe shapes.

## Conclusions

Among the plantar intrinsic and extrinsic foot muscles, the ADDH-OH primarily contributes to TFS production. The ACSA_max_ and MV of the ADDH-OH alone explained 42 and 29%, respectively, of the variance in TFS.

## Data Availability

Please contact the corresponding author for data request.

## Abbreviations

ABDM: Abductor digiti minimi
ABH: Abductor hallucis
ACSA: Anatomical cross-sectional area
ACSA_max_: Maximal anatomical cross-sectional area
ADDH-OH: Adductor hallucis oblique head
ADDH-TH: Adductor hallucis transverse head
FDB: Flexor digitorum brevis
FDL: Flexor digitorum longus
FHB: Flexor hallucis brevis
FHL: Flexor hallucis longus
MRI: Magnetic resonance imaging
MV: Muscle volume
PCSA: Physiological cross-sectional area
QP: Quadratus plantae
TFS: Toe flexor strength
